# Editorial: Viral Encephalitis

**DOI:** 10.3389/fmicb.2020.599257

**Published:** 2020-11-11

**Authors:** Mei-Ling Li, Bo-Shiun Chen, Shin-Ru Shih

**Affiliations:** ^1^Department of Biochemistry and Molecular Biology, Rutgers-Robert Wood Johnson Medical School, Piscataway, NJ, United States; ^2^Research Center for Emerging Viral Infections, College of Medicine, Chang Gung University, Taoyuan, Taiwan; ^3^Department of Neuroscience and Regenerative Medicine, Medical College of Georgia, Augusta University, Augusta, GA, United States; ^4^Department of Laboratory Medicine, Linkou Chang Gung Memorial Hospital, Taoyuan, Taiwan; ^5^Department of Medical Biotechnology and Laboratory Science, College of Medicine, Chang Gung University, Taoyuan, Taiwan; ^6^Research Center for Chinese Herbal Medicine, College of Human Ecology, Chang Gung University of Science and Technology, Taoyuan, Taiwan; ^7^Research Center for Food and Cosmetic Safety, College of Human Ecology, Chang Gung University of Science and Technology, Taoyuan, Taiwan; ^8^Graduate Institute of Health Industry Technology, College of Human Ecology, Chang Gung University of Science and Technology, Taoyuan, Taiwan

**Keywords:** viral encephalitis, antiviral and vaccine, epidemiology and diagnosis, enterovirus, Flavivirus, herpes virus, astrovirus, Pseudorabies virus (PRV)

Viral encephalitis, an inflammation of the brain parenchyma caused by virus infection, poses a serious threat to global public health. The major causal agents worldwide are herpes viruses and arboviruses. HSV-1 is the leading cause of adult encephalitis in developed countries. While Japanese encephalitis is the most common encephalitis worldwide, West Nile virus is the most widespread virus. Recently, Zika virus, chikungunya virus, and Dengue virus have co-circulated in many regions of the Americas (Silva, 2013; Acevedo et al., 2017; Venkatesan and Murphy, 2018).

Despite the advance in studying the complex interplay between viruses and infected cells, the pathogenesis of viral encephalitis is largely unknown. For example, the regulation of blood brain barrier (BBB) permeability and the regulation of immune responses to virus infection in the CNS remain to be explored.

This Research Topic combines 10 publications from 64 authors, including 3 review articles and 7 research articles, covering many aspects of RNA and DNA virus-associated encephalitis.

The two in-depth reviews by Chen B-S et al. and Majer et al. provide readers a convenient way to understand the most up-to-date knowledge of non-polio, enterovirus-associated encephalitis. These two articles cover all aspects of recent advances in enterovirus-induced encephalitis including the routes of CNS infection, tropism, virulence, immune response, and molecular pathogenesis. These two reviews provide insightful information for the development of effective antiviral strategies and vaccines.

Chen J. et al. described the epidemiological and phylogenetic analysis of the very first large-scale Echovirus 30 (E-30, genus Enterovirus) outbreak in northwest China in 2015. The study shows children aged 6–15 years are more susceptible to E-30 infection. The highly similar E-30 genomes in this outbreak suggested an aggregate outbreak of E-30. The dominant lineage has a complex genetic transmission that indicates the infection initiated from coastal provinces of China and then spread to other parts of the country. The study provides valuable information for future surveillance.

Globalization promotes virus transmission and disease spreading among continents. The ongoing COVID-19 pandemic is a devastating example (Harapan et al., 2020; Yuki et al., 2020). Astrovirus-associated encephalitis cases have been reported in Asia, North American, and Europe. Giannitti et al. characterized the first case of bovine astrovirus-associated encephalitis in Uruguay (South America). Phylogeographic analysis suggests the virus was introduced to South America from Europe and later spread to North American and Japan. The study advances the understanding of the geographic distribution and genetic diversity of astroviruses.

The vast majority of papers in this Research Topic are related to Flavivirus-associated encephalitis with distant emphasis on different viruses. Rothan et al. described expression of Z-DNA-binding protein 1 (ZBP1) restricting virus replication in West Nile virus (WNV)-induced encephalitis. WNV is the leading cause of viral encephalitis in the United States. ZBP1 plays an essential role in triggering robust immune responses. WNV infection dramatically up-regulates ZBP1 expression in mouse brain and primary mouse cells. Deletion of ZBP1 results in higher morbidity and mortality after WNV infection in mice. Infection of ZBP1–/– mice with the virus is lethal, indicating that ZBP1 is required for survival after WNV infection.

Tomar et al. reported the development of a polymerase spiral reaction assay for real time detection of WNV from clinical samples. As there is no FDA-approved vaccine or antiviral against WNV, early diagnosis of WNV infection is critical for clinical management and disease control. The reverse transcription polymerase spiral reaction (RT-PSR) assay rapidly and accurately detects the envelope gene of WNV using real-time turbidimeter or visual detection by the addition of SYBR Green I dye. The assay is validated and is able to detect as low as one copy of RNA, which is 100-fold higher than conventional RT-PCR. The assay has a potential to be used for rapid testing of a large number of clinical samples.

Zika virus (ZIKV) is another important member in the Flavivirus family that causes severe neurological complications such as meningoencephalitis in adults and microcephaly in fetus (Musso and Gubler, 2016). To better understand how ZIKV crosses the placental barrier and the blood brain barrier (BBB) to cause microcephaly in a fetus, Chiu et al. used human placenta trophoblasts cells (JEG-3) and human brain-derived endothelial cells (hCMEC/D3) as *in vitro* models to study the mechanisms ZIKV uses to cross the physiological barriers. The study shows ZIKV infection changes the permeability of JEG-3 cells by disrupting the tight junction and by transcytosis. ZIKV crosses the BBB by transcytosis.

Japanese encephalitis virus (JEV) is the major cause of viral encephalitis worldwide. JEV infection targets neurons and receptor interacting serine/threonine-protein kinase 3 (RIPK3), which contributes to neuron inflammation and death. Bian et al. found that RIPK3 plays multiple roles in JEV infection. The progression of JEV was inhibited in RIPK3-knockout (RIPK3–/–) mice and in RIPK3-knockdown neuro2a cells with a significantly increased level of interferon (IFN)-induced protein 44-like gene (IFI44L). Over-expression of IFI44L decreases JEV replication in neuro2a cells and double knockdown of RIPK3 and IFI44L promotes virus replication. On the other hand, RIPK3 inhibits IFI44L expression to promote JEV propagation in neurons.

DNA virus infection can induce encephalitis as well. Equine herpesvirus-1 (EHV-1) is one of the most important pathogens of horse and causes a constant threat to the equine industry worldwide. There are increasing numbers of devastating equine herpesviral myeloencephalopathy outbreaks. Oladunni et al. reviewed the discovery of the virus, the latest developments of treatment and disease control, and summarizes recent advances in the research of EHV-1 pathogenesis. The information presented is useful for development of strategies to limit the spread of EHV-1 in equine populations.

Pseudorabies virus (PRV) is a member of the Herpevirus family. Qi et al. used a fosmid library as a platform and Red/ET recombination technology to generate recombinant PRV fused with EGFP. The rescued recombinant virus is used to monitor retrograde and anterograde moving in the axon, making it a powerful tool for neuronal circuit analysis. The study is helpful for development of vaccines against PRV and other herpesviruses.

We hope that this Research Topic provides readers a better understanding of virus-associated encephalitis. A deeper understanding of viral encephalitis will facilitate the development of innovative antiviral approaches.

## Author Contributions

All authors listed have made a substantial, direct and intellectual contribution to the work, and approved it for publication.

## Conflict of Interest

The authors declare that the research was conducted in the absence of any commercial or financial relationships that could be construed as a potential conflict of interest.
